# Supplementary material for: Evidencing leprosy neuronal inflammation by 18-Fluoro-deoxy-glucose
Source: PLoS Negl Trop Dis. 2023 Jun 5;17(6):e0011383. doi: 10.1371/journal.pntd.0011383 (PMC10270593; doi:10.1371/journal.pntd.0011383)
Supplement: Attachment — Submitted filename: Reviewer 1 Comments.docx [file pntd.0011383.s001.docx]

**Reviewer #1**

**Evidencing Leprosy Neuronal Inflammation by 18-Fluoro deoxy-glucose**

1. It is difficult to understand why the authors are recommending a high-end investigation which is expensive, not easily available, not safe and also not required.

2. It is well known in leprosy that the clinical manifestations and changes in nerve conduction appear after the nerve tissue is damaged beyond a critical level. If detected at this stage- it only indicates a better prognosis- as compared to a patient who demonstrates nerve deficit on clinical examination. Why then PET/CT- when it can be detected clinically- it is not helpful and not required.

3. In leprosy patients – development of neuritis as part of reaction- is not difficult to diagnose and no investigation – less so the PET/CT.

4. The authors suggest that it can help in difficult to diagnose cases- with nerve involvement-viz neural leprosy- because the invasive nerve biopsy can be avoided. Will the PET/CT confirm the diagnosis of leprosy as cause of neuritis? Biopsy is the only procedure which will give you the definitive diagnosis. Moreover, neural forms of leprosy without clinical evidence of inflammation will not be detected by the PET/CT as per the authors.

5. The PET/CT will help in distinguishing between a ‘**new’** episode of neuritis and chronic neuropathy. What does this word ‘**new**’ indicate? Moreover, an episode of symptomatic neuritis due to reaction does not require such a specialized investigation.

6. How the fibrosed and in-excitable nerves in patient No.7 and 8 showed the increased uptake of glucose? How do we expect /explain increased cellular inflammation in such a nerve? The figures provided do not show fibrosis and moreover, it is too early in the course of the disease after treatment to expect complete fibrosa.

More specifically:

7. The aim of the study is not well perceived. When the patients being investigated are established cases of leprosy- the diagnosis is made- why consider again differential diagnosis of neuropathy and investigate with nerve conduction study, nerve biopsy and 18F-FDG PET/CT and draw a blank. Where was the need?

8. Same with the other 2 patients with a confirmed diagnosis of leprosy -any symptomatic nerve involvement including pain, swelling, worsening of nerve deficit will logically be considered as part of reaction. No extra or special investigation is needed.

9. Doing whole body (from cranial cap to root of the thighs) imaging with PET/CT seems to be going too far. How will this investigation help in understanding leprosy neuropathy histopathology?

Patient selection:

10. One patient was excluded because of ‘recurrent’ leprosy. What is ‘recurrent’ leprosy?

11. Why this classification of neuropathy into polyneuropathy, mononeuropathy and multiple neuropathies- knowing fully well that this classification is more so for understanding- but not exactly applicable to leprosy, because we are studying only the nerves available for examination and the pathology can be focal, patchy and involving only part of the nerve. Same applies to nerve conduction studies and compound muscle and sensory nerve action potentials- how can any study so clearly delineate axonal damage and or demyelinating lesions- when the process in leprosy is mixed and in continuum.

12. Same is true for the chronology of involvement of skin or nerves- earlier, later or together. But where is the need and what is their relevance to PET/CT study or these patients in totality.

13. Finally, the hope and wish that the degree and type of infiltrate (if known? It is already well known) in the asymptomatic neuropathy and that in the acute neuritis will help finding a new drug on this basis which will help treat the neuritis / nerve damage.

14. Four Patients had biopsies from the dorsum of the hand- it is difficult to understand how this correlation can be interpreted and how much of it can be relied upon.

15. Biopsies were taken from dorsal cutaneious ulnar nerve (n-4) and sural nerve (n-2) -nerves at these sites may not be uniformly involved and so may not give a true picture.

16. Number of patients studied is too small.
