# Supplementary material for: Evidencing leprosy neuronal inflammation by 18-Fluoro-deoxy-glucose
Source: PLoS Negl Trop Dis. 2023 Jun 5;17(6):e0011383. doi: 10.1371/journal.pntd.0011383 (PMC10270593; doi:10.1371/journal.pntd.0011383)
Supplement: Attachment — Submitted filename: To Reviewer 1 .docx [file pntd.0011383.s002.docx]

**To Reviewer #1**

We would like to thank you for your comments, which helped us to improve our text.

We would like to clarify some points that seem not to have been very evident regarding our real objective with this work, which was a research work and we had no intention of using highly complex and costly tests in the routine of leprosy investigation, but to help us understand some unknown aspects of the pathophysiology of silent neuritis.

**Evidencing Leprosy Neuronal Inflammation by 18-Fluoro deoxy-glucose**

1.It is difficult to understand why the authors are recommending a high-end investigation which is expensive, not easily available, not safe and also not required. R. This article is a research work with the aim of obtaining a better understanding of what happens in LL patients who evolve with worsening neurological function, often asymptomatic.

2. It is well known in leprosy that the clinical manifestations and changes in nerve conduction appear after the nerve tissue is damaged beyond a critical level. If detected at this stage- it only indicates a better prognosis- as compared to a patient who demonstrates nerve deficit on clinical examination. Why then PET/CT- when it can be detected clinically- it is not helpful and not required. R. The results of the clinical and neurophysiological evaluation will depend on the patient's degree of neurological impairment at that time. However, the injury process cannot be determined using these resources. The purpose of using PET was to try to demonstrate in vivo some inflammatory/metabolic process that would help us understand how the nerve is injured and whether we could offer a better treatment for these patients, since they do not respond to corticosteroids.

3. In leprosy patients – development of neuritis as part of reaction- is not difficult to diagnose and no investigation – less so the PET/CT. R. Unfortunately, this is not always true, some patients have silent neuritis, very common in LL patients, and if they are chronic patients with recurrent neuritis in the same nerve, often the clinical and neurophysiological findings are similar to the findings of patients with pain neuropathic that occurs in some patients after neuritis (chronic neuropathy as a sequela of leprosy).

4. The authors suggest that it can help in difficult to diagnose cases- with nerve involvement-viz neural leprosy- because the invasive nerve biopsy can be avoided. Will the PET/CT confirm the diagnosis of leprosy as cause of neuritis? Biopsy is the only procedure which will give you the definitive diagnosis. Moreover, neural forms of leprosy without clinical evidence of inflammation will not be detected by the PET/CT as per the authors. R. We don't think PET is a substitute for nerve biopsy. We have reformulated some sentences in the text, as perhaps our explanation was not clear to the reviewer.

5. The PET/CT will help in distinguishing between a ‘**new’** episode of neuritis and chronic neuropathy. What does this word ‘**new**’ indicate? R. As described in answer 3, some patients have recurrent neuritis, so a new episode indicates that the nerve has already suffered an episode of neuritis and subsequently (at least 3 months after the resolution of the previous one) there was a new episode. Moreover, an episode of symptomatic neuritis due to reaction does not require such a specialized investigation. R. This is not always true, some episodes of neuritis are less classic and the patient has loss of neural function without obvious signs of neuritis, the so-called silent neuritis.

6. How the fibrosed and in-excitable nerves in patient No.7 and 8 showed the increased uptake of glucose? How do we expect /explain increased cellular inflammation in such a nerve? R. The biopsied site was not exactly the site where the capture took place. It is widely recognized that the involvement of fascicles and therefore nerve fibers in leprosy occurs asymmetrically. Therefore, not all the nerve was affected very likely. The figures provided do not show fibrosis and moreover, it is too early in the course of the disease after treatment to expect complete fibrosa.

More specifically:

7. The aim of the study is not well perceived. When the patients being investigated are established cases of leprosy- the diagnosis is made- why consider again differential diagnosis of neuropathy and investigate with nerve conduction study, nerve biopsy and 18F-FDG PET/CT and draw a blank. Where was the need? R. We have made some changes to the text to make our objectives clearer.

8. Same with the other 2 patients with a confirmed diagnosis of leprosy -any symptomatic nerve involvement including pain, swelling, worsening of nerve deficit will logically be considered as part of reaction. No extra or special investigation is needed. R. For the diagnosis of these patients obviously not. However, the objective was to understand if the acute neuritis really captured in order to show that patients who did not capture probably had nerve damage by another mechanism.

9. Doing whole body (from cranial cap to root of the thighs) imaging with PET/CT seems to be going too far. How will this investigation help in understanding leprosy neuropathy histopathology?

R. This was a research project. The patients signed an informed consent form and were aware of the objectives of performing the tests in this study.

Patient selection:

10. One patient was excluded because of ‘recurrent’ leprosy. What is ‘recurrent’ leprosy?

R. These are patients who completed the entire MDT cycle, were considered cured for a few years, and became reinfected. These patients were excluded because we wanted to assess a recent diagnosis of leprosy neuropathy.

11. Why this classification of neuropathy into polyneuropathy, mononeuropathy and multiple neuropathies- knowing fully well that this classification is more so for understanding- but not exactly applicable to leprosy, because we are studying only the nerves available for examination and the pathology can be focal, patchy and involving only part of the nerve. Same applies to nerve conduction studies and compound muscle and sensory nerve action potentials- how can any study so clearly delineate axonal damage and or demyelinating lesions- when the process in leprosy is mixed and in continuum.

R. In a neurological examination, it is very important to classify the type of peripheral nerve impairment for differential diagnosis between the various etiologies of peripheral neuropathies.

12. Same is true for the chronology of involvement of skin or nerves- earlier, later or together. But where is the need and what is their relevance to PET/CT study or these patients in totality.

13. Finally, the hope and wish that the degree and type of infiltrate (if known? It is already well known) in the asymptomatic neuropathy and that in the acute neuritis will help finding a new drug on this basis which will help treat the neuritis / nerve damage.

14. Four Patients had biopsies from the dorsum of the hand- it is difficult to understand how this correlation can be interpreted and how much of it can be relied upon.

R. The dorsal cutaneous branch of the ulnar nerve is the site chosen for biopsy of this nerve, as it is a purely sensory branch. The ulnar nerve is one of the most compromised nerves in leprosy.

15. Biopsies were taken from dorsal cutaneious ulnar nerve (n-4) and sural nerve (n-2) -nerves at these sites may not be uniformly involved and so may not give a true picture. R. R. The biopsy site is predetermined by nerve accessibility and not by the location of the disease. We have anatomical limitations.

16. Number of patients studied is too small.

R. This is only a case series.
